# Characterizing the Transmission Potential of Zoonotic Infections from Minor Outbreaks

**DOI:** 10.1371/journal.pcbi.1004154

**Published:** 2015-04-10

**Authors:** Adam J. Kucharski, W. John Edmunds

**Affiliations:** 1 Centre for the Mathematical Modelling of Infectious Diseases, London School of Hygiene & Tropical Medicine, London, United Kingdom; 2 Fogarty International Center, National Institutes of Health, Bethesda, Maryland, United States; Pennsylvania State University, UNITED STATES

## Abstract

The transmission potential of a novel infection depends on both the inherent transmissibility of a pathogen, and the level of susceptibility in the host population. However, distinguishing between these pathogen- and population-specific properties typically requires detailed serological studies, which are rarely available in the early stages of an outbreak. Using a simple transmission model that incorporates age-stratified social mixing patterns, we present a novel method for characterizing the transmission potential of subcritical infections, which have effective reproduction number R<1, from readily available data on the size of outbreaks. We show that the model can identify the extent to which outbreaks are driven by inherent pathogen transmissibility and pre-existing population immunity, and can generate unbiased estimates of the effective reproduction number. Applying the method to real-life infections, we obtained accurate estimates for the degree of age-specific immunity against monkeypox, influenza A(H5N1) and A(H7N9), and refined existing estimates of the reproduction number. Our results also suggest minimal pre-existing immunity to MERS-CoV in humans. The approach we describe can therefore provide crucial information about novel infections before serological surveys and other detailed analyses are available. The methods would also be applicable to data stratified by factors such as profession or location, which would make it possible to measure the transmission potential of emerging infections in a wide range of settings.

## Author Summary

The transmission potential of a new infection depends on both the transmissibility of the pathogen and the level of immunity in the host population. However, it can be difficult to measure these properties if there are limited experimental studies of population immunity. By incorporating social contact patterns into a mathematical model of disease transmission, we show that it is possible to estimate both pathogen transmissibility and pre-existing immunity from available data on the size of outbreaks. When an infection does not transmit efficiently between humans, estimates often have to be made using case data from a limited number of small outbreaks. We find that, even with limited data, our technique can accurately evaluate the transmission potential of ‘stuttering’ chains of infection. We use the method to characterise transmission of four real infections: monkeypox, influenza A(H5N1) and A(H7N9) and MERS-CoV.

## Introduction

Infections that spill over into humans from an external reservoir have the potential to cause epidemics with substantial morbidity and mortality, particularly if there is limited pre-existing immunity in the host population [1, 2]. However, novel pathogens do not always transmit efficiently when first introduced into human populations. Outbreaks of infections such as Middle East respiratory syndrome coronavirus (MERS-CoV) [3, 4] and monkeypox [5] have generally occurred as ‘stuttering chains’ of transmission [6], generating a relatively small number of linked clusters of cases without evidence of sustained transmission. Infections such as influenza A(H5N1) [7] and A(H7N9) [8] also appear to be subcritical at present, having so far failed to transmit efficiently between humans.

To assess the risk posed by novel infections, it is important to quantify their transmission potential. Transmissibility can be summarised using the effective reproduction number, *R*, defined as the average number of secondary cases produced by a typical infectious host [9]. The reproduction number can be separated into two components: the inherent transmissibility of a pathogen, and the level of susceptibility in the host population. In some circumstances, susceptibility might be reduced as a result of pre-existing immunity from previous vaccination campaigns, as is the case with monkeypox [5, 10], or prior exposure to a similar pathogen, as has been suggested for influenza A/H1N1p [11]. Such immunity will not necessary be distributed evenly across the population: if pathogens circulate over an extended period of time, or vaccination campaigns have been discontinued, pre-existing immunity is more likely to be found in older age groups [12].

It has been shown that the size distribution of minor outbreaks can provide information about the value of the effective reproduction number [13, 14]. However, existing techniques for estimating transmission potential from outbreak size data generally represent transmission in the host population using single-type branching process [15, 16, 17, 18]. As a result, it is not possible to distinguish between inherent pathogen transmissibility and population susceptibility. For instance, a highly transmissible pathogen in a mostly immune population might have the same effective reproduction number as an infection with lower inherent transmissibility spreading between fully susceptible hosts.

To characterize both the inherent properties of the pathogen and the level of population immunity, we analysed both the size and age distribution of minor outbreaks. Individuals of different ages have heterogeneous social contact patterns and hence different risks of infection during an outbreak [19, 20, 21]. Pre-existing immunity in older age groups can alter this pattern [22], making it possible to separate the reproduction number into its pathogen- and population-specific components. We made use of this observation by developing a novel age-structured model of stuttering transmission chains, which combined reported social contact data with a multi-type branching process [23, 24].

First we derived an expression for the outbreak size distribution in an age-stratified population, in which transmission between different age groups depended on the number of physical contacts reported in the POLYMOD survey in Great Britain. Next, we used simulated outbreaks to examine whether the model could distinguish between different types of infection using only age-stratified final outbreak size data. Finally, we analysed observed outbreak data for monkeypox, influenza A(H5N1), A(H7N9) and MERS-CoV, and found that it was possible to accurately characterize pathogen transmissibility and pre-existing host immunity.

## Results

### Outbreak size distributions for age-structured populations

We explored the age pattern of infection by calculating the joint outbreak size distribution across different age groups. It has been suggested that the post-childhood drop in risky contacts that occurs around age 20 is a dominant factor shaping influenza dynamics [25], and the intense contacts between children make them an important epidemiological group for respiratory infections [26, 12]. We therefore divided the population into two groups: under 20 and over 20 year olds.

**Fig 1:**
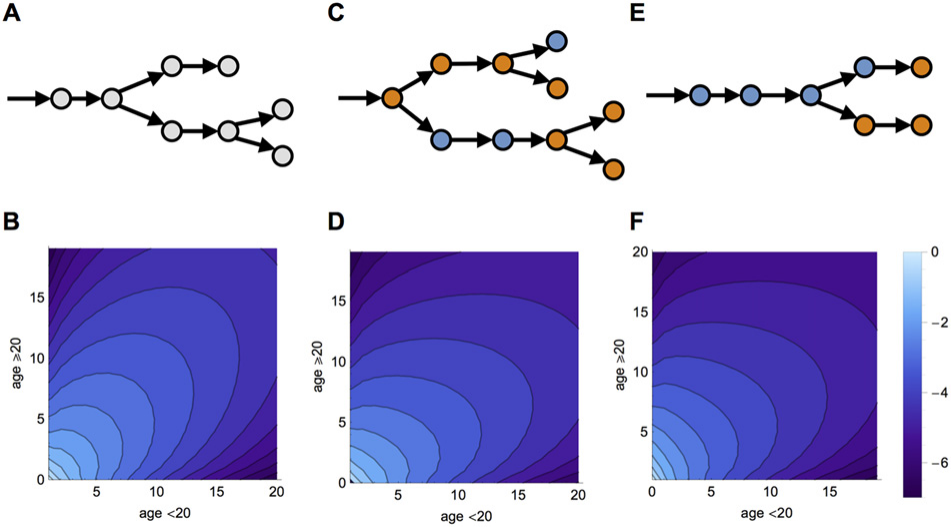
Transmission chains and joint outbreak distributions. (A) Example transmission chain when the population mixes homogeneously. (B) Joint probability an introduction will produce a transmission chain of a given size in each of the two age groups (on log_10_ scale) when the outbreak starts in the under 20 age group. (C) Transmission chain when mixing is age-dependent and infection starts in the under 20 age group. (D) Joint outbreak size distribution when model incorporates social contact data from Great Britain and infection introduced into the under 20 age group.(E) Transmission chain when infection starts in oldest age group. (F) Joint size distribution when infection starts in the over 20 age group. We assume *R*_0_ = 0.6.

In a homogeneously mixing population, all individuals generated the same mean number secondary cases in the model (Fig. 1A). When the infection was introduced into the under 20 age group, the outbreak size distribution was therefore relatively symmetric between the two groups (Fig. 1B). When the offspring distribution of secondary cases depended on reported physical contacts between different groups in the UK (S1A Fig.), this pattern changed. Each infected host could generate secondary cases in either group, and the mean number of cases generated depended on which group the infected host was in (Fig. 1C). We assumed a fully susceptible population, which meant that the average number of secondary cases generated by a typical infectious individual was equal to the basic reproduction number, *R*_0_ [9]. If infection started in the under 20 age group, there was a noticeable bias in the outbreak size distribution, with large outbreaks in under 20 year-olds more likely than large outbreaks in the over 20s (Fig. 1D). When the infection started in the over 20 age group (Fig. 1E), the offspring distribution shifted, and the probability of large outbreaks in the under 20 age group decreased (Fig. 1F).

### Identifying anomalously large outbreaks

**Fig 2:**
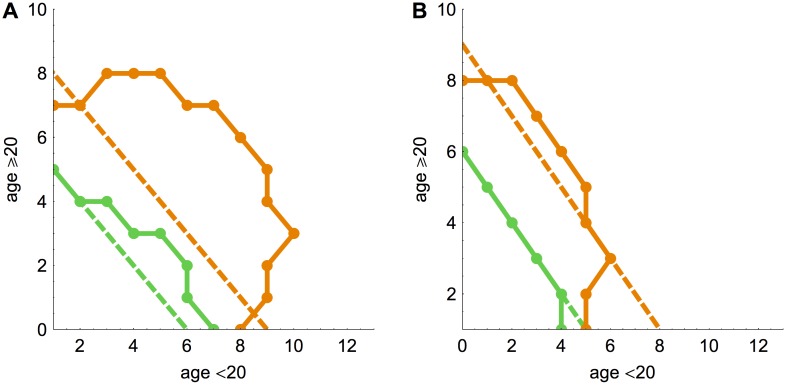
Threshold for anomalously large outbreak sizes. (A) Primary case is in the under 20 age group. Points show joint outbreak sizes that have less than 10^−3^ probability of occurring. Green points, *R*_0_ = 0.3; orange, *R*_0_ = 0.7. (B) Primary case is in the over 20 age group.

We used the outbreak size distribution to identify what constitutes an anomalously large outbreak for a particular *R*_0_. We defined this as an outbreak size that has a less than 10^−3^ probability of occurring in our model. When the infection was introduced into the under 20 age group, there was an asymmetry in the threshold for an unusually large outbreak in the UK (Fig. 2A). If *R*_0_ = 0.7, a chain of at least 8 cases was not unusual if some of the secondary cases are children, yet it is if the secondary cases are all adults. The conditions for an anomalously large outbreak shifted when infection started in the eldest group (Fig. 2B). In some cases the thresholds curved inwards. In Fig. 2A, when *R*_0_ = 0.7 an outbreak of size 7 was anomalously large if all secondary cases were in the youngest group, but an outbreak of size 10 was not unusual if between 2–8 secondary cases were in the eldest group. As the infection was introduced in the youngest group, this suggested that chains of transmission were more likely to persist if they crossed into the eldest age group. The threshold also curved inwards when the infection started in the eldest group (Fig. 2B). An outbreak of size 5 was unusual if all the secondary cases were in the youngest group, but an outbreak of size 8 was not anomalous if there were 3 cases in the eldest group. This implies that having a single case in the introductory age group and several in the other group was unlikely when *R*_0_ = 0.7. As suggested by the next generation matrix (S1A Fig.), the primary case would generally create additional cases within the same group rather than infect only individuals in the other group.

### Estimating transmissibility and pre-existing immunity

Using age stratified-data, we found that is was possible to distinguish between inherent pathogen transmissibility and pre-existing host immunity. We simulated outbreaks using a multi-type branching process with two groups, then used the outbreak size distribution to infer *R*_0_ and relative immunity in older individuals. We assumed that the under 20 age group was fully susceptible to infection, and the relative susceptibility of the over 20 age group, denoted *S*, could vary. Each outbreak was seeded randomly in the susceptible population. In the UK the under 20 age group make up 24% of the total population, so in the absence of immunity, the probability of the outbreak starting in this group was 0.24.

**Fig 3:**
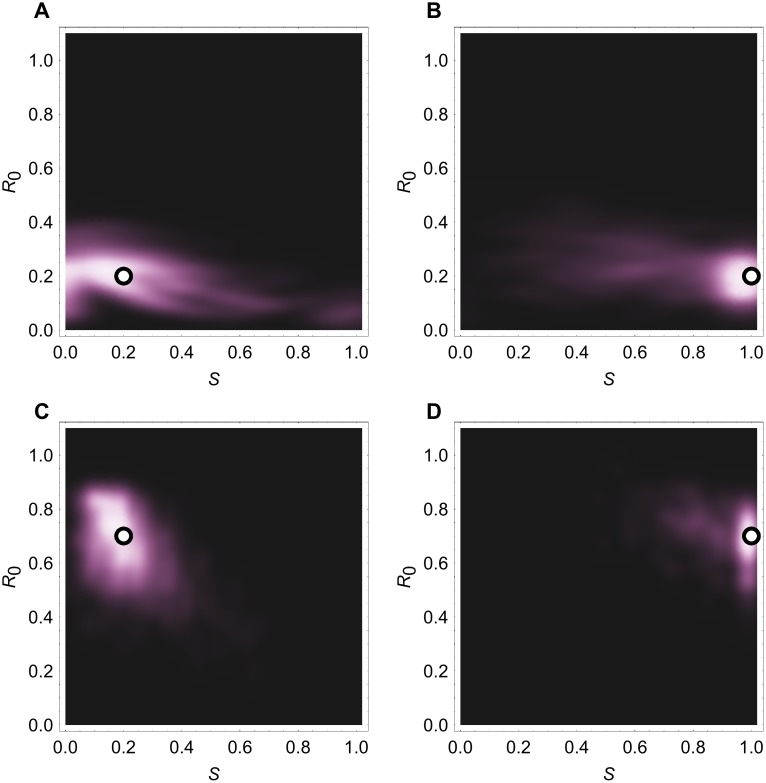
Estimates of *R*_0_ and relative susceptibility of over 20 age group, *S*, from simulated data. We simulated 1000 sets of 50 outbreaks, and found the maximum likelihood estimates (MLEs) for parameters for each set. White dots show true parameter values; heat map shows distribution of the 1000 MLEs.

To test our inference framework, we simulated four different scenarios. First, we examined two infections with the same *R*_0_ = 0.2, but different levels of immunity in the over 20 age group. In one scenario, only 20% of hosts over age 20 were susceptible to infection (i.e. *S* = 0.2); in the other, the population was fully susceptible (*S* = 1). We simulated 50 spillover events, and found the maximum likelihood estimate of *R*_0_ and *S*. We repeated this process for 1000 sets of outbreaks, obtaining reliable estimates of both *R*_0_ and *S* (Figs. 3A-B). Next, we considered the same two susceptibility values, but for an infection with *R*_0_ = 0.7. The model was again able to distinguish between the different scenarios (Figs. 3C-D). The structure of the reproduction matrix (Equation 2) means that *R*_0_ and *S* should always be identifiable in the model, given enough data, because *R*_0_ scales the entire matrix, whereas *S* only scales the transmission rate to the older age group.

We used our estimates of *R*_0_ and relative immunity in the over 20 age group to calculate the effective reproduction number. We compared these values with estimates from an inference framework based on a single-type branching process [15, 16, 17, 18]. In all four scenarios, our estimates for *R* are less biased in the age-structured model (Table 1). However, the relative sum-squared error is smaller in the single-type model when *R*_0_ is small. This is because accurate inference across the two age groups requires sampling from the tail of the joint outbreak size distribution, which is achieved either when *R*_0_ is larger (Table 1), or when more outbreak data are available. When inference is performed using data from a larger number of outbreaks, the relative error for the age-structured model is smaller than for the non-stratified framework (S2 Fig.).

**Table 1:** Accuracy of estimation of effective reproduction number, *R*.

*R*_0_	*S*	*R*	Model	*R* bias	Relative *R* error
0.2	0.2	0.16	Single-type	-0.026	0.36
			Age-structured	-0.002	0.38
0.2	1	0.2	Single-type	-0.030	0.32
			Age-structured	0.008	0.34
0.7	0.2	0.56	Single-type	-0.066	0.21
			Age-structured	-0.021	0.17
0.7	1	0.7	Single-type	-0.070	0.17
			Age-structured	-0.014	0.12

We simulated 1000 sets of 50 spillover events, and found the maximum likelihood estimates of the reproduction number, $\hat{R}$, for each set.

Regardless of the degree of transmissibility or immunity, we systemically underestimate *R* in the single-type model (Table 1). This bias is the result of our assumption that introductions occur randomly across the susceptible population, and illustrates an important caveat to inference of *R* from the mean outbreak size in a single-type branching process model. If the proportion of cases that are introduced to each age group is equal to the dominant eigenvector of the reproduction matrix, it is possible to obtain unbiased estimates for *R* using only the mean outbreak size (see Text S1). However, if the true proportion of introductions in the under 20 group is less than number of introductions implied by dominant eigenvector, we will underestimate *R* in a single-type model (S3 Fig.). Conversely, if the true proportion of introductions is larger, we overestimate *R*. In our model of transmission chains in Great Britain, we assumed a child-dominated social contact matrix but relatively flat population structure. In the absence of immunity, the probability the infection starts in the under 20 age group was therefore 0.24. However, the relevant component of the dominant eigenvector of the reproduction matrix is 0.68. If the probability of introduction is less than this—as it is in our model—the homogeneous mixing assumption will lead to an underestimate of *R* (S3 Fig.). The age structured model avoids dependency on age-specific exposure risk by accounting for which age group the infection started in when performing inference (Equation 13). If there were a disproportionate number of introductions in a particular age group, the structure of the likelihood function means that it would not bias our estimate for *R*.

We also tested whether our inference approach, which assumed social contact data reflects age-specific transmission, was sensitive to misspecification of the ‘true’ transmission process. We simulated data using different assumptions about age-specific infection rates but left the inference model unchanged. First, we simulated outbreak data using a multi-type branching process with 15 age groups. As in the inference model, transmission between different groups depended on reported physical contacts from the POLYMOD survey in Great Britain. Although the inference model only used two age groups, it correctly identified the four different combinations of transmissibility and susceptibility (S4 Fig.). Next, we simulated data using two ages groups, but with transmission based on the average number of reported physical contacts across 8 European countries in the POLYMOD study (S1B Fig.). The relative error in *R* was generally slightly larger (S1 Table), but we were still able to obtain accurate scenario estimates (S5 Fig.). When we considered a generic child-dominated next generation matrix (S1C Fig.), our estimates for *S* were more variable, but we were still able to distinguish between pathogen transmissibility and pre-existing immunity (S6 Fig.). Finally, we considered a transmission matrix in which adults were dominant (S1D Fig.). As expected in such a heavily mis-specified model, we were not able to accuracy estimate *S* and *R*_0_ (S7 Fig.).

### Application to real outbreaks

Using our age-stratified framework, we characterized the transmission potential of four infections (Table 2): influenza A(H5N1); influenza A(H7N9); Monkeypox; and MERS-CoV. As we could not be certain that the under 20 age group was fully susceptible, we did not infer the basic reproduction number, *R*_0_. Instead, we defined *ρ* to be the effective reproduction number when both groups were equally susceptible (i.e. *S* = 1). If in reality the under 20 age group had no immunity to the infection then *ρ* = *R*_0_. For our analysis of MERS and monkeypox outbreak data, we used the average reported physical contacts from POLYMOD across 8 European countries (S1B Fig.). For H5N1 and H7N9, we used physical contact data from Southern China (S1E Fig.).

**Table 2:** Details of outbreaks analysed.

Infection	Location	Period	Cases	Clusters	Source
Monkeypox	Central Africa	24/08/70–30/10/79	47	41	[5]
Influenza A(H5N1)	Indonesia	21/07/05–22/11/13	192	176	WHO, [43]
Influenza A(H7N9)	Shanghai & Jiangsu	19/02/13–30/04/13	59	55	[44]
MERS	Globally	20/09/12–01/08/13	110	42	[4]

Clusters denote sets of human cases that have a known epidemiological link; single cases are defined as clusters of size one.

**Fig 4:**
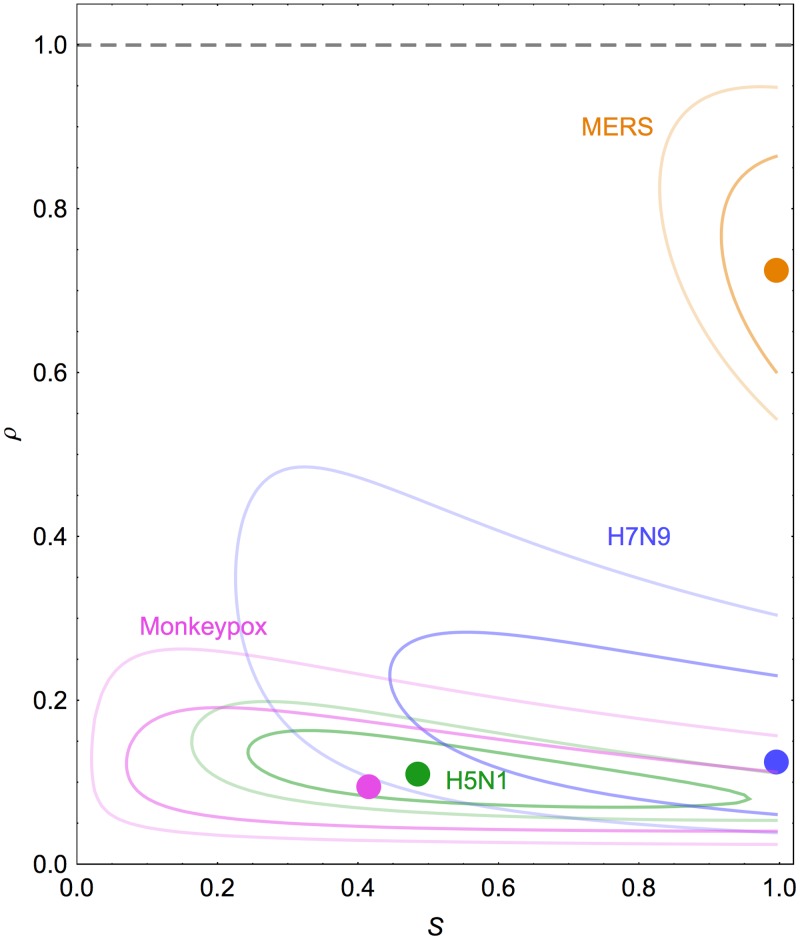
Characterization of transmission potential from observed outbreak size distributions. Each point shows joint maximum likelihood estimate of the effective reproduction number if both age groups were equally susceptible, *ρ*, and the relative susceptibility of over 20s, *S*. Dark line indicates 80% confidence interval (CI); light line is 95% CI. Blue, influenza A(H7N9); green, influenza A(H5N1); pink, monkeypox; orange, MERS.

We measured transmission potential by jointly inferring *ρ* and *S* for each of the four infections. Our maximum likelihood estimates suggest that the over 20 age group had substantial pre-existing immunity against monkeypox and H5N1, and no immunity against H7N9 or MERS-CoV (Fig. 4). These estimates agree with values derived from detailed studies of vaccination and infection history (Table 3). We could not perform such a comparison for MERS-CoV, however, as we could find no studies reporting measurements of population-level immunity for humans.

**Table 3:** Comparison of our parameter estimates and previously published estimates of *R*, from studies using single-type models, and *S*, from studies of vaccination history [5] and seroprevalence [28, 29] and models of immune acquisition [27].

Infection	*R* estimate (95% CI)	*R* in literature	*S* estimate	*S* in literature
Monkeypox	0.08 (0.02–0.22)	0.09 [5]	0.42 (0.02–1)	0.4–0.7* [5]
H5N1	0.10 (0.05–0.17)	0.14 (0.004–0.39) [7]	0.48 (0.15–1)	0.36 (0.30–0.44)* [27]
H7N9	0.12 (0.04–0.42)	0.10 (0.01–0.49) [45]	0.99 (0.22–1)	≈1 [28, 29]
MERS	0.73 (0.54–0.96)	0.63 (0.49–0.85) [3, 4]	0.99 (0.82–1)	–

*See Text S1 for details of calculation.

Our estimate of *S* for monkeypox exhibited considerable uncertainty: the 95% confidence interval spanned 0.02–1. This was likely the result of the small number of clusters we analysed. To examine whether a larger number of clusters might improve our model estimates, we performed a simulation study using an infection with limited transmissibility in a population with pre-existing immunity (i.e. a similar scenario to monkeypox transmission). We simulated 50 spillover events, with *R*_0_ = 0.25 and *S* = 0.5, then attempted to infer the parameters from the age-stratified outbreak size data. We found that the 95% confidence interval of the joint distribution of *R*_0_ and *S* was very broad (S8 Fig.). However, when we simulated 150 or 250 spillover events instead, the uncertainty in our estimates shrank, and we were able to obtain more precise parameter estimates (S8 Fig.).

Using our model, we also estimated *R* for each set of real outbreaks. Our estimates were similar to previously published estimates that assumed a single-type population. However, the confidence intervals for our estimates were generally smaller (Table 3). Influenza A(H7N9) had an effective reproduction number of 0.08 (95% CI 0.02–0.23), influenza A(H5N1) had *R* = 0.10 (0.05–0.18) and monkeypox *R* = 0.08 (0.02–0.22). Our estimate of *R* for MERS-CoV was 0.73 (0.54–0.96), whereas in a single-type branching process model *R* = 0.63 (0.49–0.85). The discrepancy was caused by the age distribution of the largest outbreak clusters. One cluster of 26 infections consisted entirely of over 20s: if transmission was indeed driven by social mixing patterns, such an outbreak would require a large *R* to persist in only one group.

During real-time analysis of an outbreak, there may be additional infections yet to be reported. It is particularly important to account for such censoring when infections are near the *R* = 1 boundary [17]. To test the robustness of our estimates for MERS-CoV when outbreak size data were censored, we extended our inference framework to account for incomplete outbreaks (methods in Text S1). When censoring was included, our estimate for *R* increased slightly to 0.77 (0.57–1.03), but our maximum likelihood estimate for *S* remained the same.

## Discussion

Obtaining accurate estimates of transmission potential is crucial for effective surveillance and control of infectious diseases. However, for emerging infections estimates often have to be made using case data from a limited number of small outbreaks. Using a multi-type branching process, we developed an inference framework to make better use of age-structured outbreak size data.

Our results show that when disease transmission is driven by social contacts between different age groups, knowledge of the age distribution of cases makes it possible to separate the effective reproduction number into two components: inherent pathogen transmissibility, and pre-existing immunity in older age groups. Based on observed outbreak size distributions, we estimated that individuals over age 20 had susceptibility to monkeypox reduced by a factor 0.4 compared with younger hosts. This value agrees well with published estimates of population susceptibility (Table 3), with cross-immunity coming from the smallpox vaccination campaigns that ended in the two decades preceding the outbreaks [5]. We also found evidence of pre-existing immunity to influenza A(H5N1) in older individuals; it has previously been suggested that such immunity could result either from prior exposure to H5N1, or from cross-immunity from previous infection with influenza A(H1N1) [27]. In contrast, we estimated that both age groups had similar levels of susceptibility to MERS and influenza A(H7N9). Given that immunity from vaccination and natural infection tends to increase with age, this suggests that there was little pre-existing immunity to these pathogens. While serological studies have found no evidence of pre-existing immunity to H7N9 virus in these locations [28, 29], serological analysis remains challenging for novel coronaviruses such as MERS-CoV [30]. The approach we describe can therefore provide crucial information about the degree of population susceptibility before serological surveys are available.

We also used our model to identify thresholds for anomalously large outbreaks. In a single-type branching process framework, the threshold is a single number: the total size of the outbreak [31, 16]. In an age-structured model, however, the threshold depends on outbreak size in each age group. The age breakdown of cases can therefore provide additional information about what constitutes an unusual outbreak which would not be available with only overall outbreak sizes. Moreover, the shape of the thresholds in Fig. 2 suggest that the infection must pass between age groups to persist. Such dynamics could be important in understanding how pathogens adapt to a new host or invade a new population, and could be explored further in future using the models we have described here.

We made several assumptions in our model. First, we assumed that secondary cases are drawn from a geometric distribution with mean *R* (or *R*_*ij*_ in the two group model). This is akin to assuming that recovery times are exponentially distributed in the standard SIR model. Other studies have assumed that the offspring distribution for secondary cases follows a negative binomial distribution, and have suggested that an increased level of over-dispersion is often appropriate when modelling disease emergence [16, 14]. However, some of this over-dispersion is captured implicitly our model as a result of the variation that comes from including social contact structure. Given appropriate data, it would be interesting to see whether individual variation in transmission can be explained by social behaviour rather than processes such as virus shedding. This would have implications for how the over-dispersion parameter should be interpreted in an age-structured framework.

We also assumed that transmission events are independent, and did not consider depletion of susceptible hosts during an outbreak. This simplification is reasonable for infections with a small effective reproduction number, but depletion of susceptibles would need to be accounted for if *R* were close to 1 [16]. In addition, we assumed that transmission potential between age groups was captured entirely by social contacts. Because we used simulated data to infer parameters, and hence had knowledge of the true model, we were also assuming that these contacts were reported accurately. We tested the accuracy of parameter estimation when the transmission process was mis-specified, and found that it was still possible to distinguish between different scenarios as long as transmission matrices in both the simulation and inference models were dominated by intense mixing between children. This is a reasonable assumption, as it has been suggested that such mixing patterns drive observed outbreaks of respiratory infections [21, 25, 32].

Although published contact matrix data were not available for Central Africa, where monkeypox cases were reported, preliminary results from social contact survey in Uganda suggest that age mixing patterns are qualitatively similar to those found in the POLYMOD study, with a clear pattern of assortative mixing between different age groups, and children reporting a larger number of contacts relative to adults (Olivier Le Polain de Waroux, personal communication).

Our work extends existing techniques for inferring epidemiological parameters from the distribution outbreak sizes. By accounting for the age structure of a population, we show that it is possible to obtain unbiased estimates of the reproduction number, and distinguish between pathogen transmissibility and immunity from outbreak size data. During an outbreak, cluster data may be difficult to obtain; cases are typically reported as aggregated totals by health ministries and WHO [33]. Our results illustrate the value of making higher resolution outbreak data available, with cluster information and covariates such as age reported along overall case numbers.

There are situations in which it could be necessary to distinguish inherent pathogen transmission potential from immunity. For example, if a vaccination campaign that protects against an infection is to change, or be discontinued, it would be important to understand how the pathogen could transmit in a fully susceptible population. This question motivated early studies of monkeypox transmission [34]. However, in studies of monkeypox outbreaks it was relatively straightforward to identify a case’s smallpox vaccination history, because the smallpox vaccine—which provided cross-immunity to monkeypox—left a distinctive scar. The same might not to be true for other vaccines.

Our methods are not limited to age structure, and could be used to examine a variety of population stratifications. Depending on the pathogen, transmission rates may also depend on factors such as profession or setting (for example, hospital versus community transmission). With appropriately stratified outbreak data, it would be possible to infer relative immunity and transmissibility in range of different groups. While spillover infections such as avian influenza and MERS-CoV are a natural application for our approach, population structure could also influence the dynamics of transmission chains following introduction via other routes. For example, novel pathogen strains could emerge via resistance-conferring mutations [35] or adaptation to a human host [36], or be introduced to a population through air travel [37]. By collecting secondary information such as the age distribution of cases, and combining these data with models such as the one outlined here, it should be possible to develop a better understanding of stuttering chains of infection and their transmission potential. During an outbreak, our framework would also be able to generate estimates of epidemiological parameters from a commonly available data source, and hence characterize transmission risk before serological surveys and other detailed analyses are available.

## Methods

### Data

Contact data came from the POLYMOD study, a diary-based survey conducted in Europe [20], and a study of social mixing patterns in Southern China [38]. In both studies, participants reported the age of their contacts on a specified day, defined as either a face-to-face conversation in the physical presence of another person, or physical skin-to-skin contact. In our simulation study, we used data on reported physical contacts from the POLYMOD survey in Great Britain (S1A Fig.) to define the level of transmission between different age groups, as there is evidence that this type of contact is better proxy for respiratory pathogen transmission than total contacts [25, 32]. Similar qualitative mixing patterns can be found in other European countries (S1B Fig.) and Southern China (S1E Fig.), as well as Southeast Asian countries such as Vietnam [39] and Hong Kong [25]. Outbreak size distributions for different infections were calculated from reported cases (Table 2). In the influenza A(H5N1) data, it was not always clear whether an outbreak cluster was seeded by a single primary case—with all other infections secondary—or multiple co-primary cases. We made the conservative assumption that each cluster had only one primary case: our estimate for *R* can therefore be considered to be an upper bound on potential transmissibility given available outbreak size data.

### Next generation matrix

We used the next generation matrix to describe the average number of secondary cases in a population with two age groups. To model age-dependent infection, we defined *m*_*ij*_ to be the mean number of contacts with individuals in age group *i* reported by participants in age group *j*, and *λ* to be the maximal eigenvalue of the matrix **M** with entries *m*_*ij*_. Defining *S* to be the relative susceptibly of group 2 compared to group 1, the average number of infections to group *i* from group *j* was therefore given by [40]:

$$\begin{array}{c} R_{ij}=\left\{\begin{array}{cc} qm_{ij}/\lambda & \text{if}\;i=1\\ qSm_{ij}/\lambda & \text{if}\;i=2 \end{array}\right. \end{array}$$ (1)

where *q* is a scaling factor depending on inherent pathogen transmissibility (i.e. *R*_0_). We defined the next generation matrix, **R**, to be the matrix with entries *R*_*ij*_,

$$\begin{array}{c} R=\left(\begin{array}{cc} R_{11} & R_{21}\\ R_{12} & R_{22} \end{array}\right)\;. \end{array}$$ (2)

The effective reproduction number of the infection, *R*, was equal to the dominant eigenvalue of this matrix. If the population was fully susceptible, then *R* was equal to the basic reproduction number, *R*_0_. If *S* = 1, but we did not know whether the population as a whole was fully susceptible, then we defined the dominant eigenvalue to be *ρ*.

### Offspring distribution

We used a multi-type branching process to model secondary infections (see Text S1 for details). Given two different types of individuals, the generating function for the offspring distribution of individual *i* was

$$\begin{array}{c} h_{i}\left(s_{1},s_{2}\right)=\sum_{j_{1}=0}^{\infty }\sum_{j_{2}=0}^{\infty }p_{s_{1},s_{2}}s_{1}^{j_{1}}s_{2}^{j_{2}} \end{array}$$ (3)

where *p*_*s*_1_, *s*_2__ was the probability that an infectious individual of type *i* generated *s*_1_ secondary cases of type 1 and *s*_2_ cases of type 2. We assumed that stochasticity in transmission was represented by a Poisson process, and that the individual offspring distribution followed a negative binomial distribution [14]:

$$\begin{array}{c} h_{i}\left(s_{1},s_{2}\right)={\left(1+\frac{R_{1i}}{k}\left(1-s_{1}\right)\right)}^{-k}{\left(1+\frac{R_{2i}}{k}\left(1-s_{2}\right)\right)}^{-k}\;. \end{array}$$ (4)

It was possible to separate this probability generating function into two components,

$$\begin{array}{c} h_{i}\left(s_{1},s_{2}\right)=g_{1i}\left(s_{1}\right)g_{2i}\left(s_{2}\right)\;. \end{array}$$ (5)

Extending approaches used for a single-type population [16], we could specify the probability that a certain number of cases of type *i* are generated by infectives of type *j* (see Text S1 for details):

$$\begin{array}{c} \mathbb{P}\left(z\;\text{cases}\;\text{of}\;\text{type}\;i\;\text{generated}\;\text{by}\;n\;\text{cases}\;\text{of}\;\text{type}\;j\right)=T_{ij}^{n}\left(z\right) \end{array}$$ (6)

$$\begin{array}{c} =\frac{1}{z!}\frac{d^{z}}{ds^{z}}{\left[g_{ij}\left(s\right)\right]}^{n}{|}_{s=0}\;. \end{array}$$ (7)

Inserting the relevant part of Equation 4 into Equation 7, we obtained

$$\begin{array}{c} T_{ij}^{n}\left(z\right)=\frac{\prod_{w=0}^{z-1}\left(kn+w\right)}{z!}{\left(\frac{R_{ij}}{k}\right)}^{z}{\left(1+\frac{R_{ij}}{k}\right)}^{-z-kn}\;. \end{array}$$ (8)

Note that in this paper we set *k* = 1. This was equivalent to assuming that recovery times were exponentially distributed, as in the standard SIR model.

### Outbreak size distribution

We used the offspring distribution to calculate the probability that an outbreak results in the following outcome: *n* total cases in group 1; *m* total cases in group 2; *a*_12_ infections in group 1 caused by infective hosts in group 2; and *a*_21_ infections in group 2 caused by infective hosts in group 1. There were two situations to consider. If *m* = 0, then *a*_12_ = *a*_21_ = 0 and hence [23],

$$\begin{array}{c} P\left(n,m\right)=\frac{T_{11}^{n}\left(n-1\right)T_{21}^{n}\left(0\right)}{n}\;. \end{array}$$ (9)

If *m* > 0, we had [24],

$$\begin{array}{ccc} P(n,m,a_{21},a_{12}) & = & \frac{a_{21}T_{11}^{n}\left(n-a_{12}-1\right)T_{21}^{n}\left(a_{21}\right)T_{12}^{m}\left(a_{12}\right)T_{22}^{m}\left(m-a_{21}\right)}{nm}\;. \end{array}$$ (10)

Finally, we used Equations 9–10 to calculate $r_{n,m}^{1}$, the probability the infection will cause an outbreak of size *n* in group 1 and *m* in group 2, given that the initial case was in group 1:

$$\begin{array}{c} r_{n,m}^{1}=\sum_{a_{12}=0}^{A_{2}}\sum_{a_{21}=0}^{m}P\left(n,m,a_{21},a_{12}\right) \end{array}$$ (11)

where

$$\begin{array}{c} A_{2}=\left\{\begin{array}{cc} 0 & \text{if}\;m=0\\ n-1 & \text{else} \end{array}\right.\;. \end{array}$$ (12)

By symmetry, we can obtain an analogous expression for $r_{n,m}^{2}$.

### Inference

If $N_{n,m}^{i}$ was the number of chains that start in group *i* and resulted in *n* cases in group 1 and *m* cases in group 2, then by Equation 11 the likelihood of parameter set *θ* given data *X* was:

$$\begin{array}{c} L\left(\theta \;|\;X\right)=\prod_{m=1}^{\infty }\prod_{n=1}^{\infty }{\left(r_{n,m}^{1}\right)}^{N_{n,m}^{1}}{\left(r_{n,m}^{2}\right)}^{N_{n,m}^{2}}\;. \end{array}$$ (13)

When only the total number of cases in a cluster was known, and not the age distribution, we instead inferred the reproduction number from the overall outbreak size distribution [16]. If *N*_*n*_ was the number of chains of size *n*, and *r*_*n*_ was the probability a transmission chain has size *n*, the likelihood function was:

$$\begin{array}{c} L\left(\theta \;|\;X\right)=\prod_{j=1}^{\infty }r_{n}^{N_{n}}\;. \end{array}$$ (14)

We obtained maximum likelihood estimates for *θ* = {*R*_0_, *S*} by calculating the two-dimensional likelihood surface and using a simple grid-search algorithm to find the maximum point. For a higher dimensional model, it might be necessary to use an alternative technique, such as Markov chain Monte Carlo [41], to ensure robust and efficient parameter estimation. Confidence intervals were calculated using profile likelihoods: for each value of *R*_0_, we found the maximum likelihood across all possible values of *S*; the 95% confidence interval was equivalent to the region of parameter space that was within 1.92 log-likelihood points of the maximum-likelihood estimate for both parameters [42].

### Performance metrics

It was not possible to obtain a tractable expression for the maximum likelihood (ML) estimates of *ρ* and *S*, and hence *R*, using Equation 13. Instead we calculated the ML estimate of the reproduction number, $\hat{R}$, using the numerically estimated maximum likelihood values for *ρ* and *S*. We used two metrics to assess the accuracy of $\hat{R}$: the estimator bias and relative error [15]. Having generated *M* sets of outbreak data using the same *R*, and found ${\hat{R}}_{i}$ for each set *i*, the estimator bias was

$$\begin{array}{c} \delta_{1}=\lim_{M\to \infty }\frac{1}{M}\sum_{i=1}^{M}{\hat{R}}_{i}-R\;, \end{array}$$ (15)

and the root mean square relative error was given by:

$$\begin{array}{c} \delta_{2}=\sqrt{\lim_{M\to \infty }\frac{1}{M}\sum_{i=1}^{M}{\left(\frac{{\hat{R}}_{i}-R}{R}\right)}^{2}}\;. \end{array}$$ (16)

### Mean outbreak size in two group model

Let ***μ*** denote the mean outbreak size matrix. If we denote entries of ***μ*** by *μ*_*ij*_, then ∑_*j*_ *μ*_*ij*_ is the mean outbreak size in group *i*. If the eigenvalues of the next generation matrix **R**, denoted *λ*_*i*_, are such that ∣*λ*_*i*_∣ < 1 for all *i*, we have

$$\begin{array}{c} \mu =IA+RA+R^{2}A+\ldots =\sum_{k=0}^{\infty }R^{k}A={\left(I-R\right)}^{-1}A \end{array}$$ (17)

where

$$\begin{array}{c} A=\left(\begin{array}{cc} \sigma_{1} & 0\\ 0 & \sigma_{2} \end{array}\right) \end{array}$$ (18)

and *σ*_*i*_ is the probability the primary infection was in group *i*.

## Supporting Information

S1 Fig Contact matrix data used in model. (A) Reported physical contacts in Great Britain in POLYMOD study [20], (B) Average across 8 European countries [20], (C) Example child-dominated matrix, (D) Example adult-dominated matrix, (E) Reported physical contacts in Southern China [38].
(TIFF)

S2 Fig Simulation results for error as number of chains increases. (A) *R*_0_ = 0.2 and *S* = 0.2. Blue line, relative error in maximum likelihood estimate for *R* in single-type model; red line, error in estimate for *R* in age-structured model. (B) *R*_0_ = 0.2 and *S* = 1.
(TIFF)

S3 Fig Inferred value of *R* using overall mean outbreak size (Equation 17). Blue line, population fully susceptible (*S* = 1); green line, over 20 age group have susceptibility reduced by half relative to under 20 group (*S* = 0.5). If the probability that the infection is introduced into group 1 (i.e. under 20 age group)
(TIFF)

S4 Fig Estimates of *R*_0_ and relative susceptibility, *S*, when simulation model is a multi-type branching process with 15 age groups. We simulated 1000 sets of 50 outbreaks, and found the maximum likelihood estimates (MLEs) for parameters for each set. White dots show true parameter values; heat map shows distribution of the 1000 MLEs.
(TIFF)

S5 Fig Estimates of *R*_0_ and relative susceptibility, *S*, when inference model assume GB contact patterns and simulation model uses average mixing patterns across 8 European countries (S1B Fig.). We simulated 1000 sets of 50 outbreaks, and found the maximum likelihood estimates (MLEs) for parameters for each set. White dots show true parameter values; heat map shows distribution of the 1000 MLEs.
(TIFF)

S6 Fig Estimates of *R*_0_ and relative susceptibility, *S*, when inference model assume GB contact patterns and simulation model uses generic child-dominated next generation matrix (S1C Fig.). We simulated 1000 sets of 50 outbreaks, and found the maximum likelihood estimates (MLEs) for parameters for each set. White dots show true parameter values; heat map shows distribution of the 1000 MLEs.
(TIFF)

S7 Fig Estimates of *R*_0_ and relative susceptibility, *S*, when inference model assume GB contact patterns and simulation model uses generic adult-dominated next generation matrix (S1D Fig.). We simulated 1000 sets of 50 outbreaks, and found the maximum likelihood estimates (MLEs) for parameters for each set. White dots show true parameter values; heat map shows distribution of the 1000 MLEs.
(TIFF)

S8 Fig Estimates of *R*_0_ and relative susceptibility, *S*, as number of spillover events increased. In simulations, *R*_0_ = 0.25 and *S* = 0.5. Age-specific contact patterns were based on reported physical contacts in Great Britain in POLYMOD study [20].
(TIFF)

S1 Table Accuracy of *R* estimation when inference matrix is mis-specified (Matrix in S1B Fig.). (PDF)

S1 Code Simulation and inference code. Simulation model generates stochastic multi type outbreaks from a two-class mixing matrix. The inference model generates maximum likelihood estimates of *R*_0_ and *S* from outbreak size data.
(R) Click here for additional data file.
