# Supplementary figures and images for: Characterizing the Transmission Potential of Zoonotic Infections from Minor Outbreaks
Source: PLoS Comput Biol. 2015 Apr 10;11(4):e1004154. doi: 10.1371/journal.pcbi.1004154 (PMC4393285; doi:10.1371/journal.pcbi.1004154)

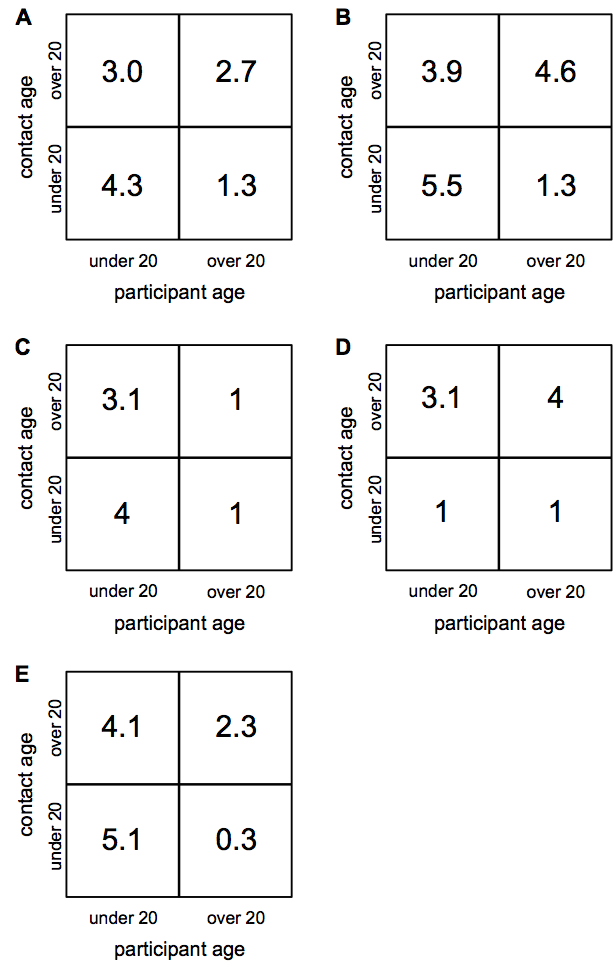

Supplement: S1 Fig — (A) Reported physical contacts in Great Britain in POLYMOD study [20], (B) Average across 8 European countries [20], (C) Example child-dominated matrix, (D) Example adult-dominated matrix, (E) Reported physical contacts in Southern China [38]. (TIFF) [file pcbi.1004154.s001.tiff]

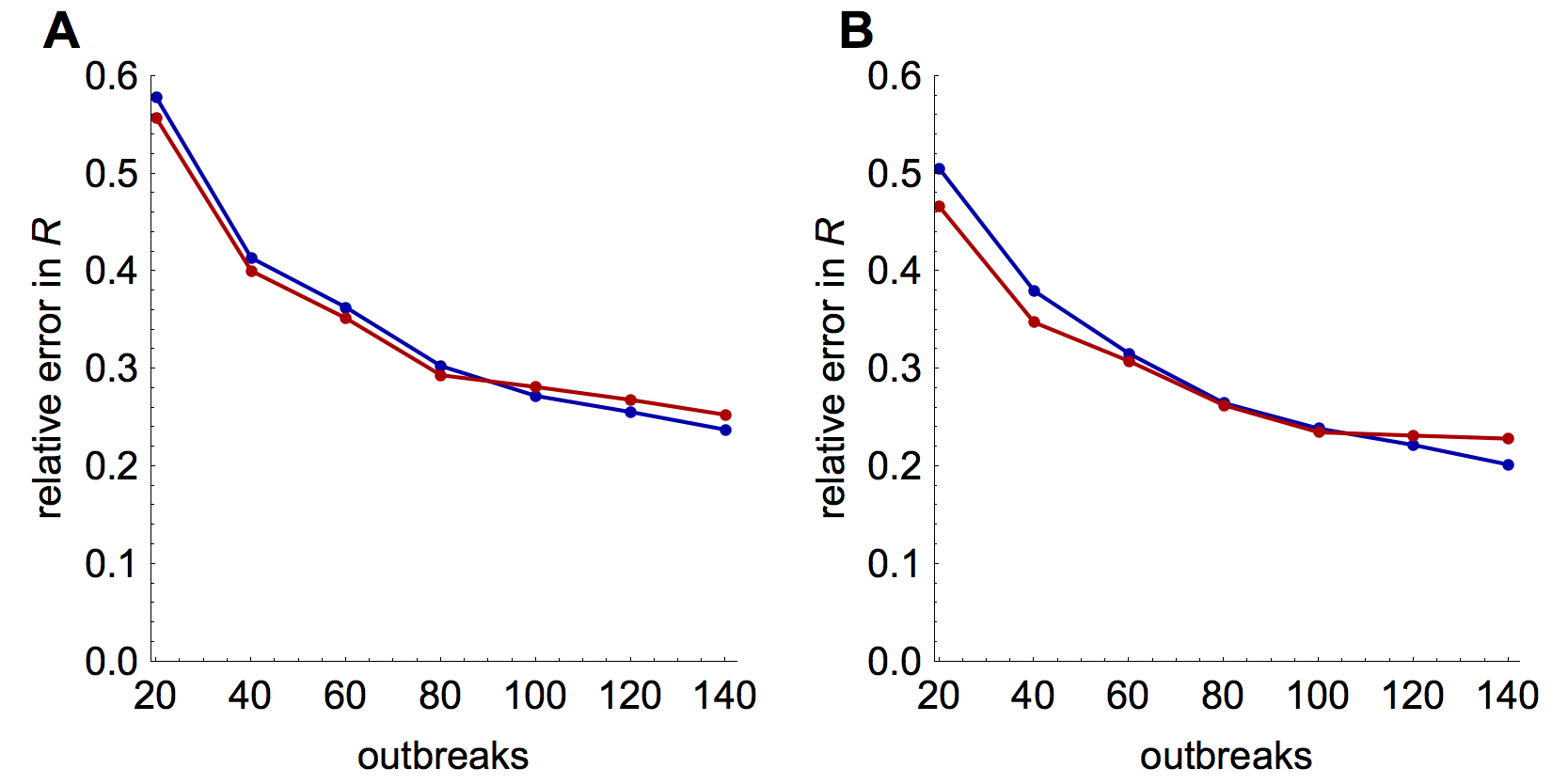

Supplement: S2 Fig — (A) R 0 = 0.2 and S = 0.2. Blue line, relative error in maximum likelihood estimate for R in single-type model; red line, error in estimate for R in age-structured model. (B) R 0 = 0.2 and S = 1. (TIFF) [file pcbi.1004154.s002.tiff]

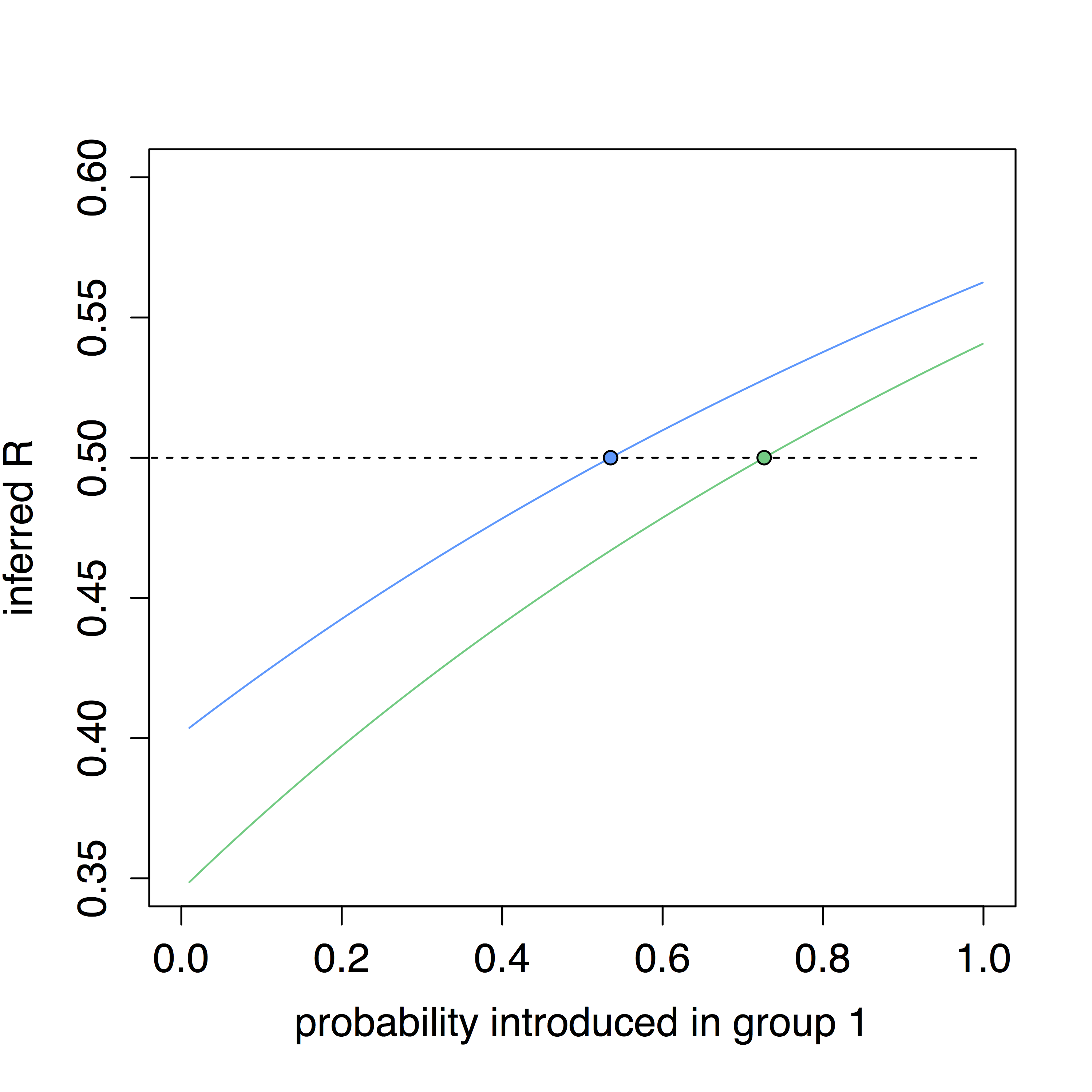

Supplement: S3 Fig — Blue line, population fully susceptible (S = 1); green line, over 20 age group have susceptibility reduced by half relative to under 20 group (S = 0.5). If the probability that the infection is introduced into group 1 (i.e. under 20 age group) (TIFF) [file pcbi.1004154.s003.tiff]

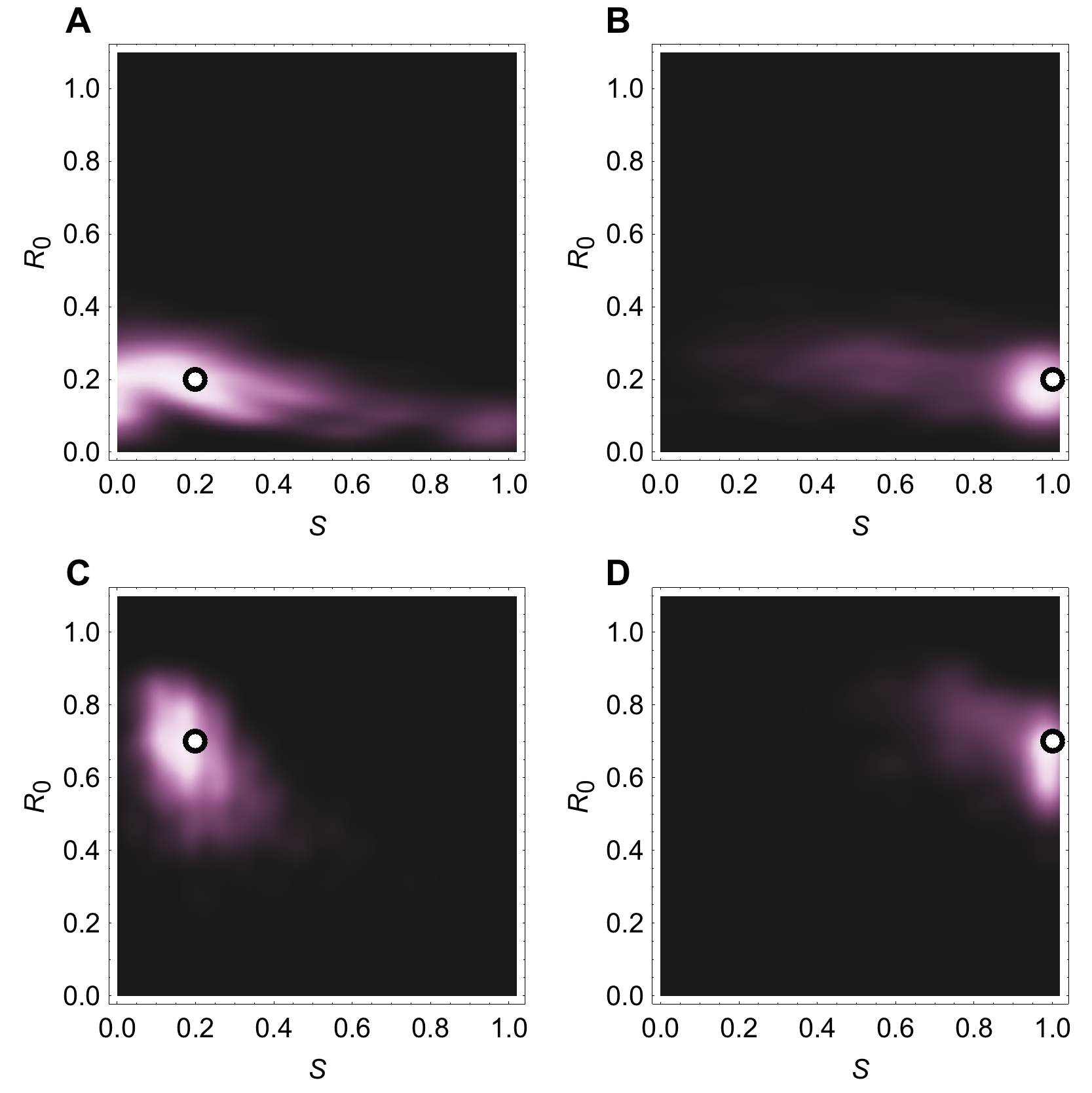

Supplement: S4 Fig — We simulated 1000 sets of 50 outbreaks, and found the maximum likelihood estimates (MLEs) for parameters for each set. White dots show true parameter values; heat map shows distribution of the 1000 MLEs. (TIFF) [file pcbi.1004154.s004.tiff]

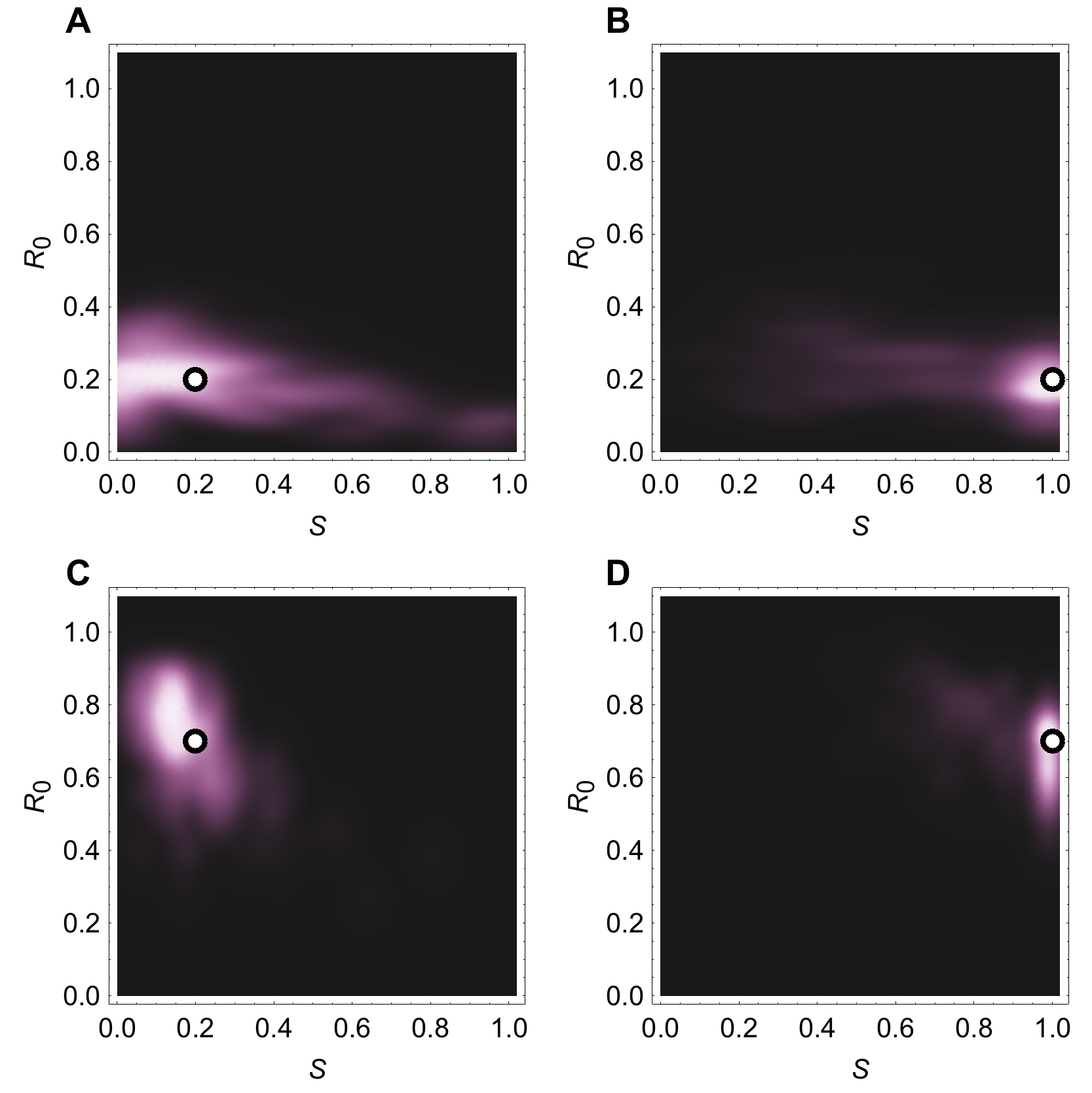

Supplement: S5 Fig — We simulated 1000 sets of 50 outbreaks, and found the maximum likelihood estimates (MLEs) for parameters for each set. White dots show true parameter values; heat map shows distribution of the 1000 MLEs. (TIFF) [file pcbi.1004154.s005.tiff]

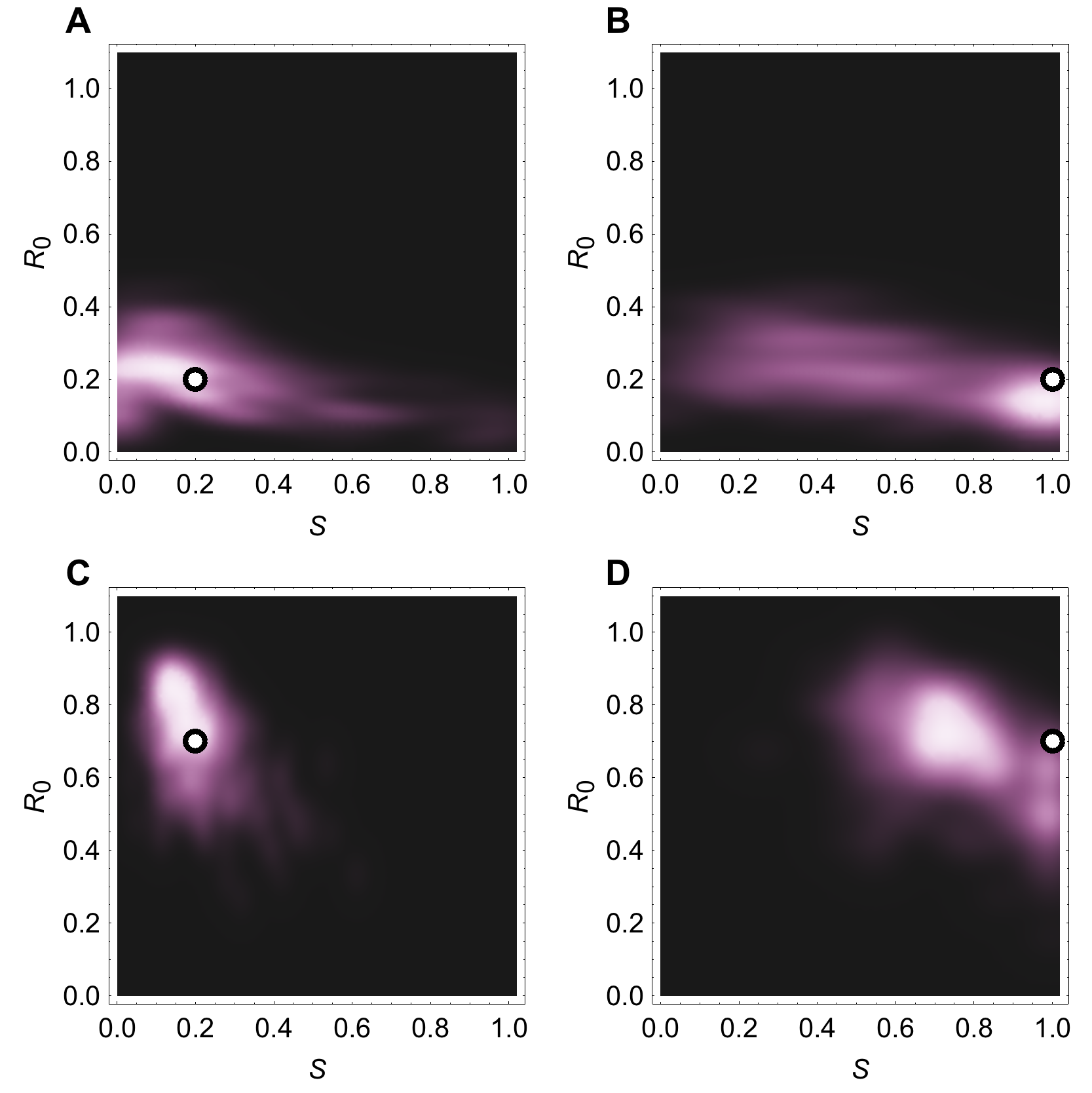

Supplement: S6 Fig — We simulated 1000 sets of 50 outbreaks, and found the maximum likelihood estimates (MLEs) for parameters for each set. White dots show true parameter values; heat map shows distribution of the 1000 MLEs. (TIFF) [file pcbi.1004154.s006.tiff]

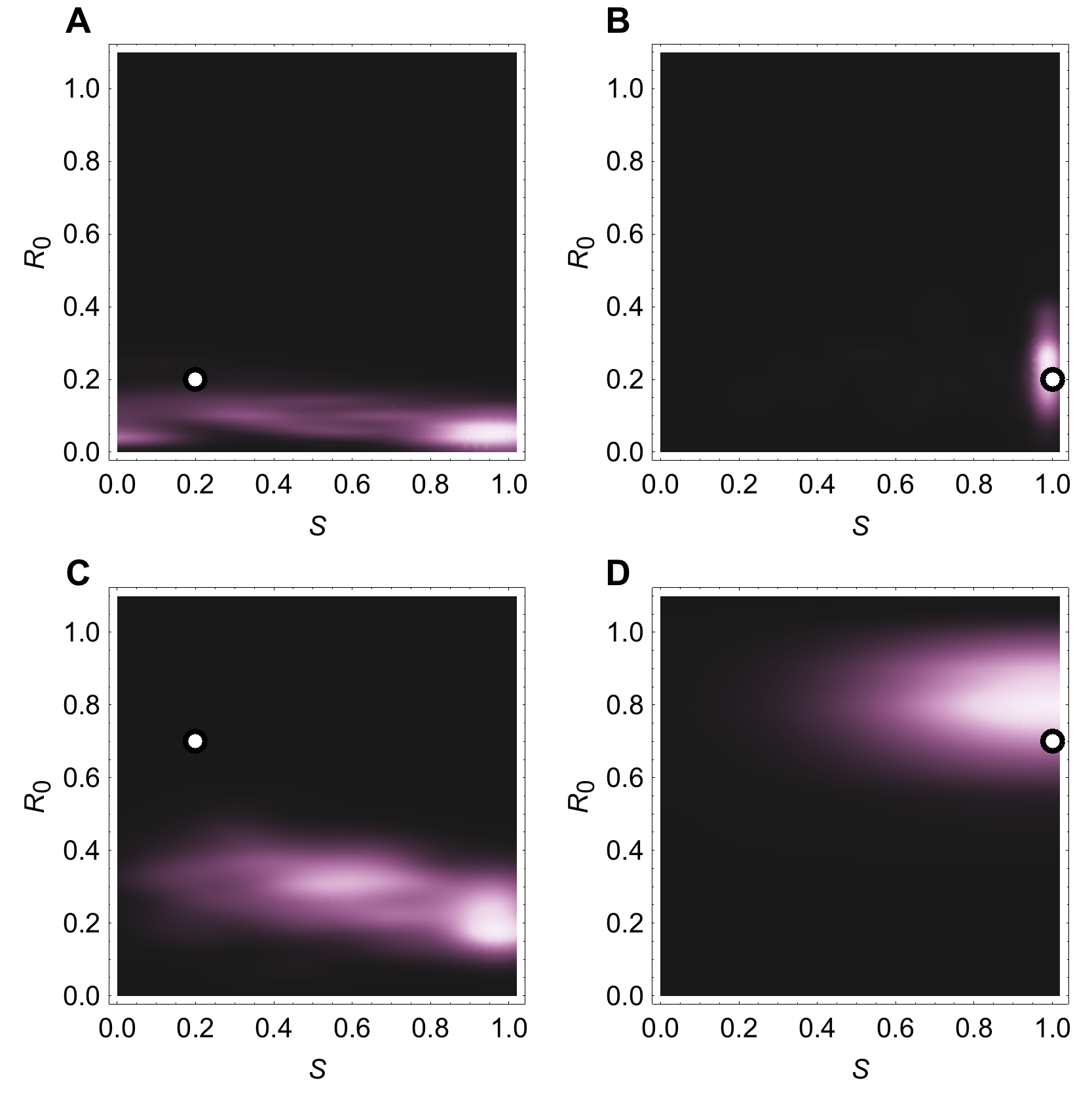

Supplement: S7 Fig — We simulated 1000 sets of 50 outbreaks, and found the maximum likelihood estimates (MLEs) for parameters for each set. White dots show true parameter values; heat map shows distribution of the 1000 MLEs. (TIFF) [file pcbi.1004154.s007.tiff]

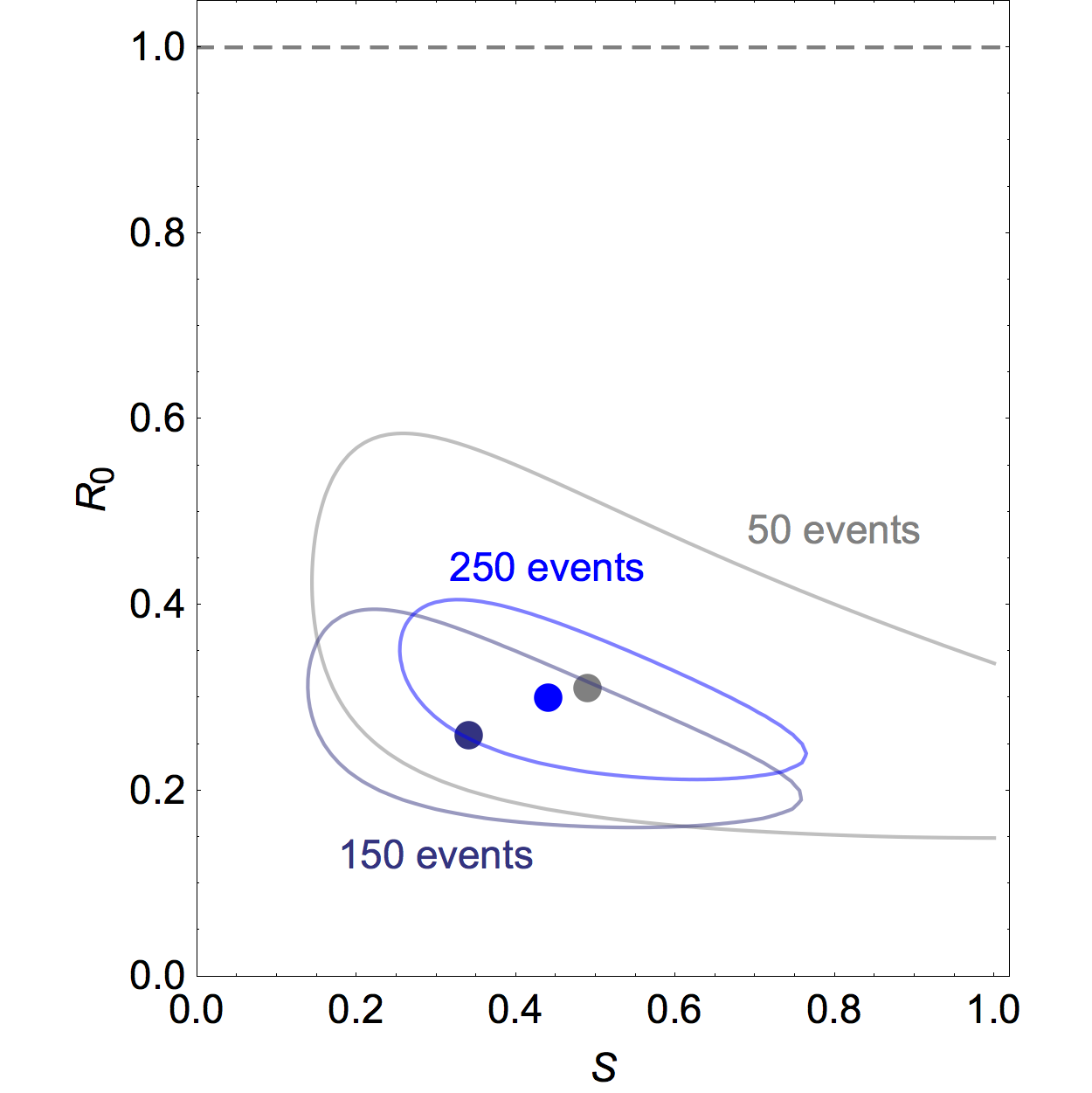

Supplement: S8 Fig — In simulations, R 0 = 0.25 and S = 0.5. Age-specific contact patterns were based on reported physical contacts in Great Britain in POLYMOD study [20]. (TIFF) [file pcbi.1004154.s008.tiff]
