# Supplementary material for: Characterizing the Transmission Potential of Zoonotic Infections from Minor Outbreaks
Source: PLoS Comput Biol. 2015 Apr 10;11(4):e1004154. doi: 10.1371/journal.pcbi.1004154 (PMC4393285; doi:10.1371/journal.pcbi.1004154)
Supplement: S1 Table — (PDF) [file pcbi.1004154.s009.pdf]

ρ	S	R	Model	R bias	Relative R error
0.2	0.2	0.16	Homogeneous	-0.025	0.38
			Age-structured	-0.004	0.39
0.2	1	0.2	Homogeneous	-0.020	0.30
			Age-structured	0.014	0.34
0.7	0.2	0.56	Homogeneous	-0.070	0.21
			Age-structured	-0.030	0.18
0.7	1	0.7	Homogeneous	-0.047	0.13
			Age-structured	-0.020	0.14
